# Queensland’s high risk foot database: tracking the length and width of Queensland’s foot ulcers

**DOI:** 10.1186/1757-1146-6-S1-O21

**Published:** 2013-05-31

**Authors:** Peter A Lazzarini, Sharon R O’Rourke, Anthony W Russell, Patrick H Derhy, Maarten C Kamp, Michael C d’Emden, Ewan M Kinnear

**Affiliations:** 1 Allied Health Research Collaborative, Metro North Hospital & Health Service, Queensland Health, Brisbane, Queensland, 4032, Australia; 2 Department of Podiatry, Metro North Hospital & Health Service, Queensland Health, Brisbane, Queensland, 4032, Australia; 3 School of Clinical Sciences, Queensland University of Technology, Brisbane, Queensland, 4059, Australia; 4 Cairns Diabetes Centre, Queensland Health, Cairns, Queensland, 4870, Australia; 5 Department of Diabetes & Endocrinology, Princess Alexandra Hospital, Brisbane, Queensland, 4012, Australia; 6 Diamantina Institute, The University of Queensland, Brisbane, Queensland, 4072, Australia; 7 Centre for Healthcare Improvement, Queensland Health, Brisbane, Queensland, 4029, Australia; 8 Department of Endocrinology and Diabetes, Royal Brisbane and Womens Hospital, Brisbane, Queensland, 4029, Australia; 9 School of Medicine, The University of Queensland, Brisbane, Queensland, 4072, Australia

## Background

Foot ulcers are a leading cause of avoidable hospital admissions and lower extremity amputations. However, large clinical studies describing foot ulcer presentations in the ambulatory setting are limited. The aim of this descriptive observational paper is to report the characteristics of ambulatory foot ulcer patients managed across 13 of 17 Queensland Health & Hospital Services.

## Methods

Data on all foot ulcer patients registered with a Queensland High Risk Foot Form (QHRFF) was collected at their first consult in 2012. Data is automatically extracted from each QHRFF into a Queensland high risk foot database. Descriptive statistics display age, sex, ulcer types and co-morbidities. Statewide clinical indicators of foot ulcer management are also reported.

## Results

Overall, 2,034 people presented with a foot ulcer in 2012. Mean age was 63(±14) years and 67.8% were male. Co-morbidities included 85% had diabetes, 49.7% hypertension, 39.2% dyslipidaemia, 25.6% cardiovascular disease, 13.7% kidney disease and 12.2% smoking. Foot ulcer types included 51.6% neuropathic, 17.8% neuro-ischaemic, 7.2% ischaemic, 6.6% post-surgical and 16.8% other; whilst 31% were infected. Clinical indicator results revealed 98% had their wound categorised, 51% received non-removable offloading, median ulcer healing time was 6-weeks and 37% had ulcer recurrence.

## Conclusion

This paper details the largest foot ulcer database reported in Australia. People presenting with foot ulcers appear predominantly older, male with several co-morbidities. Encouragingly it appears most patients are receiving best practice care. These results may be a factor in the significant reduction of Queensland diabetes foot-related hospitalisations and amputations recently reported.

